# Comparison of the 18-item and 6-item Lubben Social Network Scales with community-dwelling older adults in Mongolia

**DOI:** 10.1371/journal.pone.0215523

**Published:** 2019-04-18

**Authors:** Sugarmaa Myagmarjav, Denise Burnette, Frank Goeddeke

**Affiliations:** 1 Mongolian National University of Medical Sciences, Ulaanbaatar, Mongolia; 2 Virginia Commonwealth University, Richmond, Virginia, United States of America; 3 Wayne State University, Detroit, Michigan, United States of America; 4 Embry-Riddle Aeronautical University, Daytona Beach, Florida, United States of America; University of California San Francisco, UNITED STATES

## Abstract

This study compares the psychometric properties of two versions of the Lubben Social Network Scale (LSNS-18 and LSNS-6) with community dwelling older adults in Mongolia. We recruited 650 older adult in the capital city of Ulaanbaatar and the country’s four rural regions. We used the Geriatric Depression Scale (GDS-15), the Short Form 12 (SF-12) physical and mental health functioning measures, and a multi-dimensional scale of social isolation for confirmatory factor analyses of the LSNS-18 and the LSNS-6. Both versions demonstrated excellent internal consistency and intraclass correlation and both correlated in expected directions with other study measures. Only the LSNS-6 provided a good fit to the data. The LSNS-6 is a viable instrument for assessing the social networks of older adults in Mongolia. The study adds to the sparse literature on measuring social and behavioral determinants of health in resource-constrained settings characterized by aging populations and high internal migration rates.

## Introduction

Social and behavioral determinants of health (SBDs) are now central concerns of public health. [1] The World Health Organization (WHO) defines social determinants of health as “the conditions in which people are born, grow, live, work and age” and “the fundamental drivers of these conditions.” [2] These conditions serve, in turn, as the ecological contexts in which individual behaviors interact reciprocally with environmental and biologic determinants to affect health. [3] SBDs often involve overlapping, mutable risk factors that lead to poor health outcomes, including low sleep quality, depression, impaired executive function, accelerated cognitive decline, cardiovascular dysfunction, impaired immunity, altered hypothalamic- pituitary–adrenocortical activity, a pro-inflammatory gene expression profile and early mortality. [4].

Social connectedness is a powerful SBD that affects health through direct and indirect pathways throughout the life course. [5,6] Common indicators of deficits in social contacts and network size are living alone, having few social network ties, and having infrequent social contact. These indicators differ from loneliness, a subjective emotional state that reflects dissatisfaction with discrepancies in desired and actual contacts and relationships. [7] Isolation and loneliness are often uncorrelated [8, 9], yet each is associated independently with poorer health behaviors and biological risk factors [10] and with mortality. [11–12]

Social networks are conceptualized as the webs of social relationships that surround individuals and the characteristics of those ties. [13] Networks are especially salient in late life, as relationships tend to diminish and health declines. [14–17] There are presently 800 million persons aged 60 years and over worldwide, and their numbers are projected to reach 2 billion by 2050. Fewer births and longer lives mean that most older adults—and the most rapidly aging populations—are in low and middle income countries (LMIC). This sector is projected to increase more than 250% by 2050, compared to 71% in developed countries. [18]

For cultural, economic and geographic reasons, older adults in LMIC rely heavily if not solely on informal social networks for support and survival. To ensure the equitable distribution of limited resources, governments must continually adjust allotments to the changing size and needs of various age groups. To do so, they need valid, user-friendly assessment tools. [19] This study builds on a prior translation and validation of the 18-item Lubben Social Network Scale (LSNS-18) with 198 older adults hospitalized for chronic health conditions in Ulaanbaatar, the capital city of Mongolia [20]. The Mongolian version of the LSNS-18 showed excellent validity and reliability and was deemed culturally acceptable. We extend this work herein, using confirmatory factor analyses to examine the performance of the LSNS-18 with a larger, more diverse sample of community dwelling older adults in urban and rural areas and to compare the LSNS-18 with the 6-item version of the same scale (LSNS-6).

### Mongolia and its aging population

Social networks are embedded in and interact with broader social and cultural contexts. [13] Mongolia is an exemplary setting for the study of social networks in a rapidly aging and urbanizing population. With 3.1 million people dispersed over 1.6 million square kilometers, it is the world’s most sparsely populated sovereign country. Before the onset of massive rural-to-urban migration during the post-Soviet 1990s, Mongolia’s ancient nomadic history necessitated high levels of interdependency; however, 41% of the entire country’s population now lives in Ulaanbaatar and the rest either live nomadic or semi-nomadic lives or reside in *aimag* (provincial) centers or *soums* (aimag subsidiaries). Owing to its history with the Soviet Union, Mongolia has very high literacy. In 2015, the literacy rate was 98.4% for the population aged 15 years and over and 96.6 for older adults.

Demographic trends in Mongolia resemble those of other developing countries. The population remains relatively young; however, median age rose from 21.8 in 2000 to 28.3 in 2017 and is projected to reach 35.2 years by 2050, when 20.1% will be aged 60 years and over [21]. The 2009 National Strategy on Population Ageing is the first and still main multisectoral response to the changing needs of older Mongolians. Advanced age is traditionally a source of high social status, and older adults are integrated with and cared for by families. Almost two-thirds (64%) of older Mongolians head a household and 87% of households that include an older adult are either nuclear or extended. But the number of older adults who live alone rose 72% between 2000 and 2010. [22] Smaller family size, constricted labor markets, internal and out-migration, and changing norms that devalue older adults are raising concerns about the effects of attenuated social networks on their health and well-being [23]

## Methods and materials

### Sample selection

**Fig 1:**
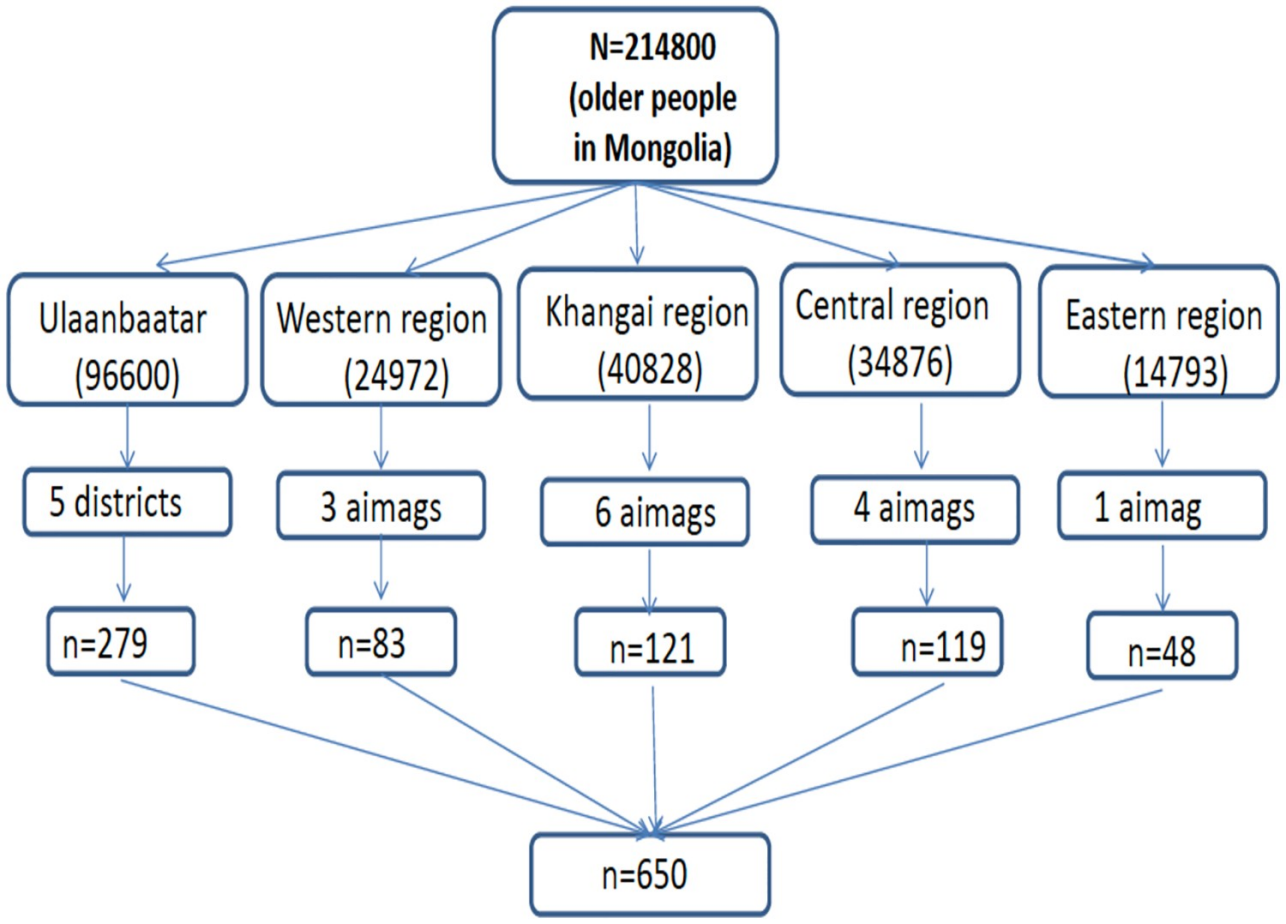
Sample selection from Ulaanbaatar and four rural regions.

The official retirement age for women and men in Mongolia is 55 years and 60 years respectively. We recruited 650 community-dwelling retirement-aged adults (411 women and 239 men) in Ulaanbaatar and four rural regions (West, Khangai, Central, and East). We identified older adults from 14 of the 21 aimags that comprise the four rural regions of the country and from 5 of the 9 districts of Ulaanbaatar (Fig 1). The gender distribution of the overall study sample reflects the proportion of men and women of retirement age in the general population.

In rural areas, we approached the Committee of Older People in each administrative unit and the appropriate unit social worker to introduce the study. The Committees then organized a local event for older adults, and a trained interviewer administered the study instrument to those who consented to participate. In Ulaanbaatar, we also approached administrative units (*khoroo*) to recruit older adults who received services or through home visits by administrative workers.

#### Data collection

Between January and October, 2013, trained interviewers used self-administered questionnaires or, when necessary, face-to-face interviews to collect data on the LSNS-18 and on sociodemographic characteristics, social support networks, health and functional status, depressive symptoms, and social isolation. There was no evidence that willingness to participate or the quality of data differed by format. Two weeks after the first administration, we re-administered the LSNS-18 with a convenience subsample of 45 respondents in Ulaanbaatar (n = 16) and 2 rural regions (n = 29). This strategy can introduce bias in the sample; however, the rest-retest reliability was sufficiently high (0.86 and 0.91, n = 45) and so similar to Cronbach’s alpha estimates (0.93 and 0.86, respectively), that with a sample of this size, it is unlikely that sampling error would significantly bias reliability estimates. Further, it is unlikely that the scale’s reliability would be a concern given such high estimates. We extracted the 6-item LSNS from the 18-item scale in order to compare the performance of the two versions.

**Lubben Social Network Scale-18: Mongolian version (LSNS-18)**. [24–25] The LSNS-18 assesses social networks with family (6 items), friends (6 items) and neighbors (6 items). Five-point Likert scales capture the size of active and intimate networks, e.g., people with whom they can talk or call on for help (0 = none, 1 = 1 person, 2 = 2 persons, 3 = 3 or 4 persons, 4 = 5 to 8 persons, and 5 = 9 or more persons) and frequency of contact and support reciprocity with network members (0 = never, 1 = seldom, 2 = sometimes, 3 = often, 4 = very often, 5 = always). Total scores are an equally weighted sum of the 18 items, ranging from 0 to 90. High scores indicate strong social networks.

Previous validation of the LSNS-18 with a hospitalized sample of older Mongolians in Ulaanbaatar [20] showed excellent internal consistency and intraclass correlations and strong convergence with social disconnectedness and perceived isolation scales and the Geriatric Depression Scale. Scores were also inversely related to self-rated health status and they discriminated well among three levels of social disconnectedness and three levels of perceived isolation. With respect to content validity, the 18 items loaded cleanly on the same three factors as the original LSNS-18, inter-factor correlations were good, all factors were correlated with the LSNS-18-M, and they accounted for two-thirds of variance in scores.

**Lubben Social Network Scale-6: Mongolian version (LSNS-6)**. The 6 items that comprise the LSNS-6 are extracted from the 18-item scale. The abbreviated version is a more parsimonious measure of the size of active and intimate networks of family and friends with whom respondents can talk or call on for help. Scores range from 0 to 30, with higher scores indicating stronger networks. English (S1 File) and Mongolian (S2 File) versions of the LSNS-6 are available as supplements to this article.

#### Other measures

**Geriatric Depression Scale ((GDS-15)** [26]. The GDS-15 is a short version of the 30-item Geriatric Depression Scale, a widely used screen for depressive symptoms in older adults. [27] A yes/no response format yields scores of 0–4 = normal; 5–8 = mild depression; 9–11 = moderate depression, and 12–15 = severe depression. The GDS has not, to our knowledge, been validated for use in Mongolia; however, a Mongolian translation of the instrument is included in the Geriatric Evaluation Guidelines approved by Ministry of health in 2010. [28]

**Short Form-12** (SF-12) [29]. The SF-12 is a subset of the Medical Outcome Study SF-36. Respondents use a Likert-type response format to rate how they have felt during the previous week in 8 domains: physical functioning (2 items), role limitations due to physical health problems (2 items), bodily pain (1 item), general health (2 items), vitality, (2 items), social functioning (2 item), role limitations due to emotional problems (2 items), and mental health, or psychological distress and psychological well-being (2 items). SF-12 scoring algorithms yield composite scores for a Physical Health Component (PHC) and a Mental Health Component (MHC), each ranging from 0 (lowest) to 100 (highest).

**Social Isolation** [30–31]. This composite scale combines multiple indicators of social connectedness and social isolation from the National Health, Social Life, and Aging Project to construct a measure that includes the two key aspects of social isolation: objective isolation, or social disconnectedness (i.e., physical separation from others) and subjective or perceived isolation (i.e., feelings of loneliness and a lack of social support). Eight items assess two dimensions of objective isolation: poor social networks (social network size, social network range, proportion of alters living in the home and average frequency of interaction with network members) and low participation in social activities (number of friends and three measure social participation (attend meetings of an organized group, socialize with family and friends, and volunteer). Subjective isolation is assessed with a 7-item scale, also with two dimensions: lack of social support (open up to family, rely on family, open up to friends, and rely on friends) and loneliness (feel you lack companionship, feel isolated, and feel left out).

Following Cornwell and Waite [30], we first standardized variables in the scales, as they used different metrics. We then averaged scores, reverse coding indicators of objective isolation so that they assess isolation rather than connectedness Objective isolation scores ranged from –3.7 to 2.60, with a weighted mean of 0.001 (SD = 0.50). Subjective isolation was assessed with a 7-item scale, also with two dimensions: lack of social support (open up to family, rely on family, open up to friends, and rely on friends) and loneliness (feel you lack companionship, feel isolated, feel left out). Subjective isolation scores ranged from –2.55 to 4.30, with a weighted mean of 0.0 (SD = 0.57). On both scales, higher scores mean greater isolation.

### Ethical Approval

The Mongolian National University of Medical Sciences bioethics committee approved the study (#2012-10/1А). All older adults who participated did so voluntarily and all participants provided written informed consent.

### Statistical analysis

We used confirmatory factor analyses (CFA) to test the factor structures of the LSNS-18-and the LSNS-6 using the lavaan package version 0 [32] in R version 3.4.4 [33]. CFA is a structural equation modeling technique for testing the measurement of latent and observed variables. [34] It is commonly used to validate the psychometric properties of measures. [35] We used multiple indices to assess the adequacy of model fit: comparative fit index (CFI), root mean square error of approximation (RMSEA), and standardized root mean square residual (SRMR). Criteria for three model fit indices were set as follows: CFI greater than .95, RMSEA close to or smaller than .06, and SRMR less than .08 [36] We also tested the measurement invariance of the LSNS-6 scale between the rural and urban (Ulaanbaatar) groups. (CFI) between models of 0.01 or less [37].

## Results

Table 1 presents descriptive data on the study sample. Just over 60% of study participants were female, and about the same proportion were married. The mean age was 66.5 years and almost half of the sample had completed either vocational or university education.

**Table 1:** Demographic characteristics (N = 650).

Variable	%	n
**Age** (66.5 ± 6.9)		
55–59	14.6	95
60–64	30.2	196
65–69	23.5	153
70–74	18.3	119
75+	13.4	87
**Sex**		
Male	36.2	239
Female	62.3	411
**Marital status**		
Married	61.1	392
Live with a partner	2.2	14
Divorced	3.3	21
Widowed	32.2	207
Single/never married	0.8	5
**Education**		
University	28.5	185
Vocational	20.0	130
High school	15.8	103
Secondary school	18.9	123
Primary school	14.0	91
No formal education	2.8	18
**Residency area**		
Rural	57.1	371
Ulaabaatar	42.9	279

### Reliability

Cronbach’s *α* was 0.93 for the LSNS-18 (subscales were 0.89 (family), .90 (friends) and .90 (neighbors)). For the total LSNS-6, *α* = .86, and for the subscales, 0.82 (family) and 0.87 (friends). ICC for test-retest was 0.86 for the LSNS-18 and 0.91 for LSNS-6.

#### Construct validity

The LSNS-18 CFA model with three correlated latent variable subscales (friends, neighbors, family) was fitted to the data. This model has 6 items, with identical stems, in each of 3 subscales. The fitted model allowed all of the error terms with similar item stems across the three subscales to correlate, with the exception of one tuple, items 1, 7, and 13. For example, the error terms for items 2, 8, and 14 were allowed to correlate in this CFA model. Theory would allow the similar item stems from the same item stems to correlate from both latent variables (family, friends), but this would create a model identification problem in our data [38] Therefore, the errors from all similar items tuples with the exception of items 1, 7, and 13 were allowed to correlate. This model resulted in a poor fit (χ^2^ = 1039.627, *df* = 120 p = .000, CFI = .885, RMSEA = .110, RMSEA 90% C.I. (.104, .117), SRMR = .055, n = 628).

**Fig 2:**
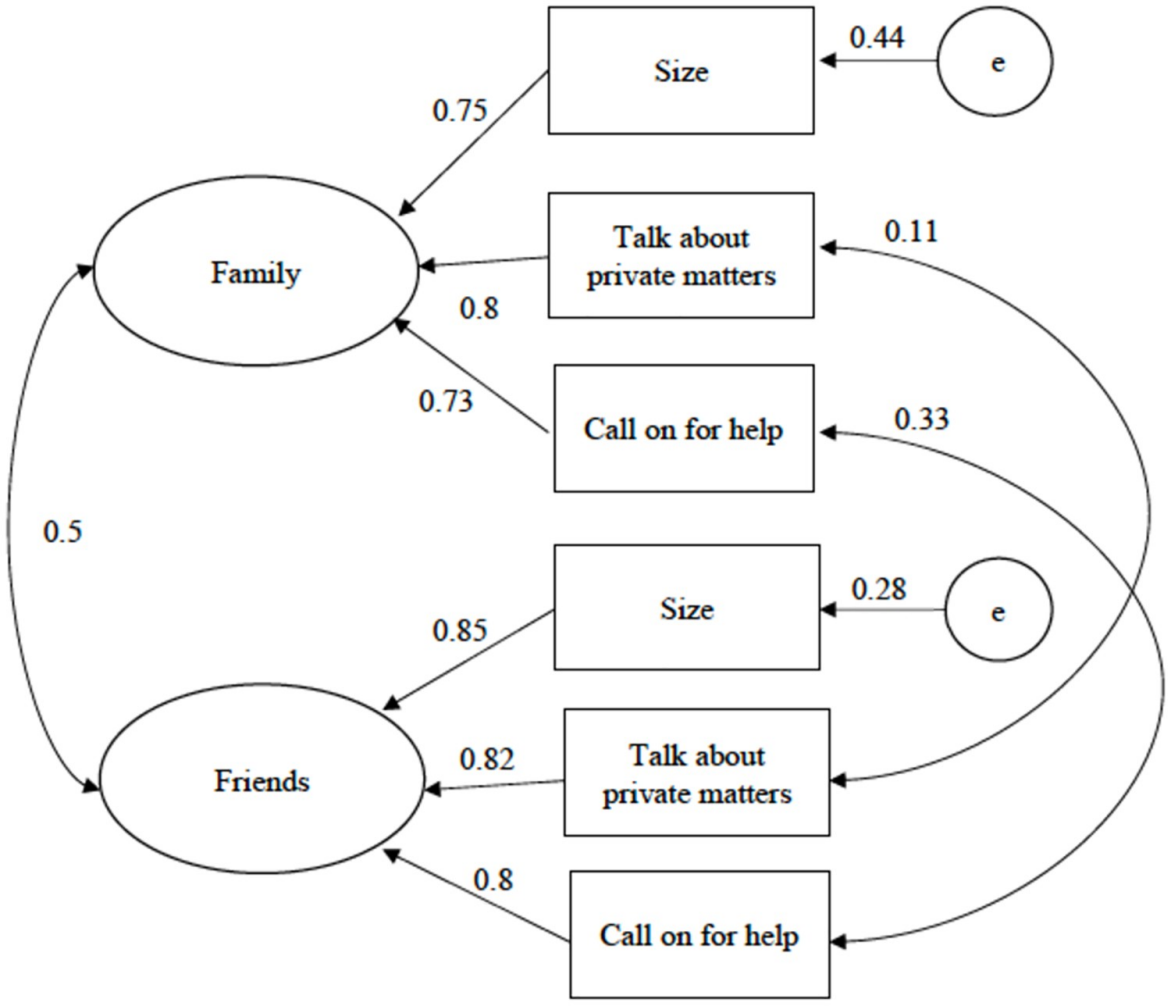
Path diagram for the LSNS-6.

The LSNS-6 CFI model with two correlated latent variable subscales (friends, family) was then fitted to the data. This model, a subset of the LSNS-18 items, uses three items for each subscale (item numbers 1, 3, 4 in friends, and items 13, 15, and 16 in family, with the same 3-item stems for each subscale. The fitted model allowed the error terms for the identical item stems of items 3 and 15 and items 4 and 16 to correlate. Although theory would allow all the similar item stems from all items to correlate, in our case this would create a model identification problem with Condition C. [38] Therefore, the errors from only two item pairs (items 3 and 15, and items 4 and 16) were allowed to correlate. This model resulted in a good fit (χ^2^ = 5.553, *df* = 6, p = .475, CFI = 1.000, RMSEA = 0.000, RMSEA 90% C.I. = [0.000, 0.049], SRMR = 0.013, n = 643). Fig 2 shows the path diagram of the model with standardized coefficients, and Tables 2 and 3 present a covariance matrix of the variables used and standardized and unstandardized path coefficients, and standard errors, respectively.

**Table 2:** Covariance matrix for LSNS-6 items for CFA.

Item	*lsns_1*	*lsns_3*	*lsns_4*	*lsns_13*	*lsns_15*	*lsns_16*
lsns_1	2.195	1.398	1.291	0.857	0.604	0.801
lsns_3	1.398	2.194	1.481	0.955	0.780	0.922
lsns_4	1.291	1.481	2.598	0.984	0.699	1.188
lsns_13	0.857	0.955	0.984	2.230	1.291	1.544
lsns_15	0.604	0.780	0.699	1.291	1.550	1.257
lsns_16	0.801	0.922	1.188	1.544	1.257	2.098

n = 643 using listwise deletion

**Table 3:** Standardized and unstandardized coefficients for LSNS-6 CFA.

Item	Latent Variable	β	B	S.E.
lsns_1	Family	0.75	1.000	
lsns_3	Family	0.85	1.142	0.062
lsns_4	Family	0.73	1.056	0.061
lsns_13	Friends	0.85	1.000	
lsns_15	Friends	0.82	0.812	0.035
lsns_16	Friends	0.84	0.960	0.040

#### Measurement invariance

One aim of this study is to validate the LSNS-6 scale for use in both urban and rural populations. The next step in this process was thus to assure measurement invariance across urban and rural samples. We used the traditional 4 step process to assess configural invariance, metric invariance, scalar invariance, and strict invariance. As the chi-square value is subject to model misspecification errors when the sample size is large, the delta CFI < 0.01 was used to test models. [37] Results are presented in Table 4 The delta CFI changes are all less than .01, meaning that the model is measurement invariant.

**Table 4:** Tests for measurement invariance between urban (N = 277) and rural (N = 277) groups.

*Model*	*χ*^*2*^	*d*.*f*.	*CFI*	*RMSEA*	*Δχ*^*2*^	*d*.*f*.	*ΔCFI*
Configural	11.98	12	1.000	0.000			
Loadings	26.69	16	0.994	0.046	14.71	4	0.006
Intercepts	33.01	20	0.003	0.045	6.32	4	0.001
Means	40.29	22	0.990	0.051	7.27	2	0.003

#### Convergent validity

LSNS-18 scores were correlated with Social Disconnectedness (r = –0.49, p *<* 0.001) and Perceived Isolation (r = –0.59, p *<* 0.001) and with the GDS-15 (r = –0.25, p *<* 0.001), all in expected directions. The LSNS-18 was positively associated with the PHC (r = .12, p *<* 0.01) and MHC subscales (r = 0.26, p *<* 0.01) of the SF-12.

Likewise, LSNS-6 scores were correlated with Social Disconnectedness (r = –0.52, p *<* 0.001) and Perceived Isolation (r = –0.54, p *<* 0.001) and with the GDS-15 (r = –0.27, p *<* 0.001) in expected directions. The LSNS-6 was also positively associated with the SF-12 PHC (r = .16, p *<* 0.01) and MHC subscales (r = 0.26, p *<* 0.01).

#### Discriminant validity

Mean scores on the LSNS-18 differed among the three levels of the Social Disconnectedness scale (F (2, 627) = 68.97, p *<* 0.001) and the three levels of the Perceived Isolation scale (F (2, 623) = 127.47, p *<* 0.001). Post hoc Tukey’s HSD tests were significant (p *<* 0.01) for all pairwise comparisons for both scales. The difference between LSNS-18 scores for the low, medium and high levels of both scales was significant.

Mean scores on the LSNS-6 differed for the three levels of Social Disconnectedness scale (F (2, 642) = 84.06, p *<* 0.0001) and the three levels of Perceived Isolation scale (F (2, 638) = 97.69, p *<* 0.0001). Post hoc Tukey’s HSD tests were significant (p *<* 0.01) for all pairwise comparisons for both scales. The difference between LSNS-6 scores for the low, medium and high levels of both scales was significant.

To summarize, unlike the LSNS-18 model, the LSNS-6 model fit the data well. The empirical factor structure appears similar to the conceptual factor structure, providing additional evidence of construct validity. The internal factor structure results, combined with the reliability and validity results, instills confidence in the use of the LSNS-6 for public health practice and academic research. Furthermore, our findings of measurement invariance suggest this model is appropriate for use with rural and urban populations.

## Discussion and conclusions

The purpose of this study was to evaluate two versions of the LSNS for use with older adults in Mongolia. We began by noting growing interest of social and behavioral determinants of health-related outcomes for public health policy and practice. The rapid aging of populations in resource-constrained LMIC heightens the urgency to develop interventions and implement policies that are grounded in local language and culture and empirically supported. Validated instruments are essential to assessment, monitoring and evaluation of public health prevention and intervention strategies.

A previous study used exploratory factor analysis with a smaller sample of hospitalized older adults in Ulaanbaatar to validate a Mongolian language version of the LSNS-18 [20]. We extend and qualify those findings by using CFA to examine this version and the 6-item version of the scale with a larger sample of community-dwelling older adults in urban and rural regions of the country. Although the sample size and analysis strategies differ in the initial and current studies, the psychometric properties of the LSNS-18 were highly commensurate. [20] Our findings add to the limited body of validation studies on measures of social and behavioral determinants of health among older adults in LMIC in the following ways.

First, we examined a well-validated, widely used measure of social networks, which is a strong social determinant of health outcomes in older adults. The combination of unprecedented rates of aging and urbanicity in many LMIC are changing traditional patterns of social life in ways that increase the risk of ruptured social networks and ultimately poor health outcomes for older people. Our findings add to the growing recognition that effective, targeted interventions require a great deal more, and more rigorous, attention to measurement issues within and across systems and countries. The LSNS-6 requires minimal administration time, which helps to ensure high response rates. Incorporating this brief measure into standard health data collection could improve care in community clinics by enhancing clinicians’ understanding of older adults’ social and behavioral risks and facilitating targeted interventions. At a systems level, the next step is to connect needs to resources that reduce risks and link improvements with health outcomes [39–40] This require systematic use of valid measures across all sectors of society, including marginalized groups that are especially susceptible to disrupted social networks. Lindskog [41] contends that despite its provision of universal, accessible and essential primary health care services, Mongolia’s health care system is unable to accommodate the health needs of poor urban in-migrants and nomadic herders in remote provinces.

Second, we used CFA to examine the LSNS-18 and the LSNS-6, and found that the LSNS-6 fit the data well. The empirical factor structure appears to be similar to the conceptual factor structure, providing additional evidence of construct validity. The internal factor structure, together with other strong psychometric properties supports use of the LSNS-6 in public health practice and academic research in Mongolia. The better fit observed for the LSNS-6 is consistent with other studies. Using CFA to examine different versions of the LSNS, Hong, Casado and Harrington [42] found the LSNS-6 to a better fit with their data on older Korean Americans. Other studies support use of the LSNS-6 with Chinese [43], Japanese [44], Portuguese [45] Mexicans and Mexican Americans [46] and samples of older adults in England, Germany, and Switzerland [47].

The LSNS-6 may perform well across a range of settings because its brevity may be advantageous in older adult populations that are less accustomed to social surveys. It addition, the LSNS-6 does not ask about neighbors. It may be more difficult to define, interact with, and report about relationships with neighbors than with more enduring relationships with family and friends. This situation may especially pertain in low-density areas of countries such as Mongolia.

A third important contribution of this study is the finding of invariance of the LSNS-6 in rural and urban populations. Given stark differences in the organization of social life and in the needs and resources available to these groups, it is surprising that few psychometric studies of older adults’ in LMIC examine this feature. The broad applicability of the LSNS-6 increases its feasibility and utility for assessing and monitoring changes in social networks country-wide.

As there is no gold standard to measure the social networks of older adults, we were not able to establish sensitivity, specificity, and positive and negative predictive values. Lubben et al. [47] suggest 12 as a clinical cut point for the LSNS-6, meaning that on average an older adult would report fewer than two people in their network. It will be important to collect systematic data on the scale’s use in Mongolia in order to advance knowledge on the adequacy of the instrument for research and practice and to establish appropriate cut point(s) for these uses. Item response theory approaches offer a promising avenue for future studies. [48]

## Supporting information

S1 File Lubben Social Network Scale (LSNS-6) English. (PDF)

S2 File Lubben Social Network Scale (LSNS-6-M) Mongolian. (PDF)
