# Supplementary material for: Comparison of the 18-item and 6-item Lubben Social Network Scales with community-dwelling older adults in Mongolia
Source: PLoS One. 2019 Apr 18;14(4):e0215523. doi: 10.1371/journal.pone.0215523 (PMC6472776; doi:10.1371/journal.pone.0215523)
Supplement: S1 File — (PDF) [file pone.0215523.s001.pdf]

Lubben Social Network Scale – 6 item (LSNS-6)
English Version

FAMILY: *Considering the people to whom you are related, either by birth or marriage . . .*

1. How many relatives do you see or hear from at least once a month?

None	One	Two	3 or 4	5-8	9 or more
0	1	2	3	4	5

2. How many relatives do you feel at ease with that you can talk about private matters?

None	One	Two	3 or 4	5-8	9 or more
0	1	2	3	4	5

3. How many relatives do you feel close to such that you could call on them for help?

None	One	Two	3 or 4	5-8	9 or more
0	1	2	3	4	5

FRIENDSHIPS: *Considering all of your friends, including those who live in your neighborhood . . .*

4. How many of your friends do you see or hear from at least once a month?

None	One	Two	3 or 4	5-8	9 or more
0	1	2	3	4	5

5. How many friends do you feel at ease with that you can talk about private matters?

None	One	Two	3 or 4	5-8	9 or more
0	1	2	3	4	5

6. How many friends do you feel close to such that you could call on them for help?

None	One	Two	3 or 4	5-8	9 or more
0	1	2	3	4	5
