# Supplementary material for: Comparison of the 18-item and 6-item Lubben Social Network Scales with community-dwelling older adults in Mongolia
Source: PLoS One. 2019 Apr 18;14(4):e0215523. doi: 10.1371/journal.pone.0215523 (PMC6472776; doi:10.1371/journal.pone.0215523)
Supplement: S2 File — (PDF) [file pone.0215523.s002.pdf]

НИЙГМИЙН ХАРИЛЦААНЫ СҮЛЖЭЭГ ҮНЭЛЭХ

ЛЮБИНИЙ АСУУЛГА (LSNS-6M)

ГЭР БҮЛ: Энд таны болон хадмын талын төрөл садан хамаарна.

1. Танд сардаа уулздаг юм уу эсвэл холбоо барьдаг хэдэн ах дүү, төрөл садангийн хүн байдаг вэ?

огт байхгүй	ганц нэгхэн	хоёр	3 - 4	5 - 8	9 ба түүнээс дээш
0	1	2	3	4	5

2.Та ах дүү, төрөл садангийнхаа хэдэн хүнтэй хувийн асуудлаа илэн далангүй ярилцаж чаддаг вэ?

огт байхгүй	ганц нэгхэн	хоёр	3 – 4	5 - 8	9 ба түүнээс дээш
0	1	2	3	4	5

3.Тусламж хүсэхэд ирэх ойр дотны хэдэн төрөл садан байна вэ?

огт байхгүй	ганц нэгхэн	хоёр	3 – 4	5 - 8	9 ба түүнээс дээш
0	1	2	3	4	5

НАЙЗ НӨХӨД: Тантай зэргэлдээ, ойр аж төрдөггүй найз нөхөд тань хамаарна.

4. Та сардаа уулзах юм уу эсвэл холбоо барьдаг хэдэн найз нөхөдтэй вэ?

огт байхгүй	ганц нэгхэн	хоёр	3 - 4	5 - 8	9 ба түүнээс дээш
0	1	2	3	4	5

5. Найз нөхдөөсөө хэдтэй нь хувийн асуудлаа ярих хэмжээний дотно байдаг вэ?

огт байхгүй	ганц нэгхэн	хоёр	3 - 4	5 - 8	9 ба түүнээс дээш
0	1	2	3	4	5

6.Тусламж хүсэхэд ирэх ойр дотны хэдэн найз нөхөд байдаг вэ?

огт байхгүй	ганц нэгхэн	хоёр	3 - 4	5 - 8	9 ба түүнээс дээш
0	1	2	3	4	5
